# Moral Disengagement as an Explanatory Factor of the Polyivictimization of Bullying and Cyberbullying

**DOI:** 10.3390/ijerph16132414

**Published:** 2019-07-07

**Authors:** Inmaculada Fernández-Antelo, Isabel Cuadrado-Gordillo

**Affiliations:** Psychology and Anthropology Department, University of Extremadura, 06071 Badajoz, Spain

**Keywords:** polyvictimization, bullying, cyberbullying, moral disengagement

## Abstract

Background: The present study’s objectives were to: (1) Identify and analyze the prevalence of poly-victims, and (2) determine how the levels of moral disengagement and the various defence mechanisms that victims use to explain abusive behavior might function as predictors of poly-bullying. Methods: The sample consisted of 1328 participants of from 9 to 14 years old. The instruments used were two questionnaires. One allows the prevalence of bullying and cyberbullying victims to be identified and analyzed. The other analyses the level of moral disengagement and the defence mechanisms to which the victims resort. Results: The results showed there to be a continuity of the role of victim in off-line and on-line contexts, turning those who are subject to these situations into poly-victims. The moral disengagement of these victims was found basically to be centered at two levels—a locus of behavior, and a locus of outcomes. Conclusions: Exposure to abuse that is continuous, of different types, and coming from different contexts must be perceived as a public health problem given the lack of effective tools to combat the situations of helplessness that the polyvictims experience.

## 1. Introduction

The access to and use teenagers and children make of technological resources as well as their connection to the Internet have led to more and more of them to try to co-exist with, and sometimes even just survive, the co-occurrence of physical and virtual scenarios. This symbiosis between childhood and the use of screens has been studied in different countries with very similar results. In the UK, around 55% of children aged three–four years regularly use tablets with Internet connection, and 24% use a computer or laptop [1]. This same study indicated that the proportions increase to 67% in the case of tablets and 49% for computers or laptops in children aged five–seven years, who also begin to use smartphones regularly. At ages 8 to 11, the percentages continue to increase: 80% for tablets, 66% for computers, and 32% for smartphones [1]. Similar results have been found in North America [2] and Spain, where around 93.8% of children and teenagers from 10 to 15 years in age have access to the Internet through the different technological resources they have available to them [3]. Access to these tools from an early age is also exposing children and teenagers to dangerous situations, not only in physical contexts, but also in virtual ones, and which they often know little or nothing about. Inability to control the use children and teenagers make of the Internet and the lack of supervision by caregivers and parents are directly related to a series of negative consequences that have a major influence on the daily lives of these young people [4]. This duality of scenarios is the cause of the co-occurrence of bullying and cyberbullying situations, as well as the durability and prolongation of the aggressor and victim roles when they pass from off-line scenarios to on-line ones.

### 1.1. Polyvictimization in the Coexistence of Off- and On-Line Scenarios

The conceptualization of the polyvictimization phenomenon still presents important discrepancies concerning the number of forms of aggression suffered, and the frequency or intensity of the abuse. In this regard, some researchers consider children who indicate having suffered at least four different forms of violence during the preceding year to be polyvictims [5]. Other researchers reinforce this identification, but without specifying the number of aggressions suffered, instead grouping them under the term ‘multiple’ [6]. These definitions emphasize the inclusion of different types of victimization and the exclusion of multiple episodes of the same type of abuse. However, other studies based on the theory of traumatic stress and cumulative adversity have stopped identifying polyvictims on the basis of the sum of the violent episodes they have experienced, to focus instead on the person. Within this working current, some researchers distinguish different victimization experiences (physical abuse, assault, sexual abuse, etc.) in measuring the potential severity of harmful situations and adversities [7,8]. In the same line, others refer to polyvictimization as a recurrent form of interpersonal victimization, basing this on the theory of cumulative trauma, which emphasizes the linear relationship between the frequency and the severity of the victimization [9].

Polyvictimization is not an isolated problem or phenomenon, neither does it belong to just a certain group of countries. It has become a global problem that respects no frontiers. Its study is leading to the generation of instruments that can identify the problem and help to determine explanatory and causal variables for its prevention and intervention. Nonetheless, the lack of consensus regarding the conceptualization of the phenomenon, the use of different instruments and scales, and the diversity of the participants’ age ranges demand prudence and reserve in interpreting the prevalence data that are being reported in the literature. The determinations of polyvictim prevalences can vary in accordance with both the minimum number of victimization experiences the researchers consider for a subject to be classified a polyvictim and the timescale over which the subject suffered those abuses. With a timescale of the preceding 12 months and the minimum number of victimization experiences suffered set at two, the range of polyvictim prevalences is 20%–40% [10,11,12]. When the number of abuses is raised to four to six, the polyvictim percentage falls to 7%–9% [13,14,15]. This class of victimization experiences is qualified as “low-level polyvictimization” [13,16]. Studies that quantify polyvictimization in which the participants have suffered seven or more types of abuse qualify it as “high-level polyvictimization”, finding a prevalence in the range of 1%–5% [12,13].

However, when the timescale is extended, and “throughout life” is accepted as a temporal unit, this multiplies the prevalence of polyvictims, with approximately 30% of subjects having suffered from 7 to 10 different types of abuse, and approximately 13% of polyvictims having suffered more than 11 types of abuse [16,17,18].

With regard to the forms of victimization suffered, many studies use as instrument the Juvenile Victimization Questionnaire [19]. This covers 36 different forms of victimization, one of which is cyberbullying [11,13,16,17]. Other studies resort to different measurement instruments, but all cover different types of victimization: Physical, emotional, verbal, social, sexual, electronic, etc. [14,15].

There have been analyses of the prevalence of polyvictims in very specific samples, such as clinical cases or delinquency. Again, there is some discrepancy among the results, which reflects the lack of consensus in defining polyvictimization. While some research has placed the prevalence at around 10% [20], other work has indicated that this proportion can even reach 90% [21].

Research addressing the motives that enhance the durability of the role of victim until it becomes one of polyvictim are fundamentally based on Seligman’s theory of learnt helplessness [22], according to which it is more likely for there to be a reduced ability to respond to repeated violent situations. To deal with these situations, children and teenagers tend to use such ineffective strategies as avoidance or denial, which only increase their stress and frustration [23]. While there is still much research to be done into the coping strategies that polyvictims use to stop and overcome the abuse they suffer, some studies seem to confirm Seligman’s predictions [24]. Other work, however, has moved away from Seligman’s explanations, and has focused on seeking the causes of the exposure to multiple traumatic events in the processes of emotional dysregulation [21,25].

The age variable and the origin of the aggressions have also been addressed in research on polyvictimization. In this sense, it has been noted [26] that polyvictimization in early childhood is associated with events that take place in the context closest to the children—their home. Polyvictimization in early childhood also seems to be associated with the prolongation of this symptomatic situation through later stages of life [19]. Thus, 9 out of 10 children who experience polyvictimization during early childhood are still polyvictims later on in life [12], although the actual experience of the harm is less in children than in teenagers or adults [27]. During adolescence, polyvictimization may also be associated with events outside the family related to sexual assaults, street violence, bullying, cyberbullying, etc., with this thus being considered the age group most at risk [28]. These facts lead to a lower likelihood of their being able to establish satisfactory relationships of trust during their lives, which in turn arrests or hinders the development of their self-esteem and self-confidence [29].

### 1.2. Moral Disengagement in Victimization Processes

Moral disengagement is conceptualized within the framework of social cognitive theory of morality in which moral standards are translated into conduct through self-regulation process [30]. Moral disengagement is a cognitive mechanism that helps individuals reduce the tension created when enacted behaviors do not match personal standards and moral norms.

For decades, it has been known that moral disengagement is strongly related to bullying, and can even be a predictor of it [30,31,32,33]. The aggressors’ behavior is explained by the activation of mechanisms designed to release the tension caused by the contradictions that arise between their moral principles and their actions [34,35]. These mechanisms, that deactivate moral censorship and self-regulation with the aim of protecting self-esteem and self-concept, are grouped into four loci that allow an individual to control their behavior: Locus of the behavior, the agent of the action, the outcomes that flow from the behavior, and the recipient of the actions [30]. The aggressors’ use of these mechanisms means that they have the possibility of legitimizing their violence as being standardized behavior.

The studies that have analyzed the connection between moral disengagement, bullying, and cyberbullying do not find any differences in the conceptualization of moral disengagement when being applied in physical or in online contexts. What varies is the instrument used to access knowledge of the degree of moral disengagement. These instruments are adaptations of the Bandura questionnaire [30] to cybernetic scenarios [36]. The results indicate that, in the virtual context, cyber-aggressors seem to show moral disengagement mechanisms that are similar to those manifested by physical aggressors [37,38]. However, others workers find moral disengagement to be more consistently related to bullying than to cyberbullying, with the characteristics of the latter involving different moral mechanisms [39,40]. More recent studies warn that cyber-aggressors have a higher level of moral disengagement than the rest of those involved in this type of situation [41].

Despite the numerous studies that have addressed this topic, there has been very little work on whether these moral imbalances are also present in the figure of the victim, and whether they contribute to perpetuating the victim’s role [42,43]. Unlike the studies focused on the figure of the aggressor whose results tend to be mutually coincidental, those that also include the victims report very different results and conclusions. Not only that, but most of these latter studies deal with the dual role of victim and aggressor [44,45], with those works whose focus was entirely on the figure of the victim being exceptional cases.

Some of the research addressing the processes of moral disengagement in victims of bullying or cyberbullying explain this disengagement as consisting of the victims separating their inaction from their excusing others’ immoral conduct, thus minimizing their need to confront or stop these aggressions. In this way, victims may disengage themselves morally to justify their inaction, and even to justify the aggressions they suffer [35,46]. In the same sense, it has been noted [44] that victims of cyberbullying also resort to a search for moral justifications, and develop a special empathy towards other victims so as to mitigate their self-attacks on their own self-esteem.

### 1.3. The Study

The introduction of moral variables into studies of the prevalence of cyberbullying has provided important explanatory and causal indicators regarding the involvement of adolescents in abuse processes [35,41,47]. However, there have been very few studies addressing the influence of moral variables on victimization rates and the durability of the role of victim. In a dual context in which the coexistence of face-to-face and virtual scenarios marks new rhythms of relationship, interaction, communication, conflict, and violence, analysis of the durability of the victim role or its conversion to that of polyvictim becomes even more relevant. Given this context, the objectives of the present work were the following: (i) To identify and analyze the prevalence of polyvictims, and (ii) to determine how the levels of moral disengagement and the various defence mechanisms that victims use to justify abusive behavior can act as predictive indicators of polyvictimization. To respond to these objectives, we posited the following hypotheses:

**Hypothesis** **1** **(H1).** *The level of moral disengagement is greater in polyvictims than in victims.*

**Hypothesis** **2** **(H2).** *The mechanisms of moral disengagement that polyvictims use vary depending on the context in which they experience their victimization: off-line, on-line, or both off- and on-line.*

**Hypothesis** **3** **(H3).** *The degree of moral disengagement and the context framing the victimization experiences are predictors of polyvictimization.*

## 2. Materials and Methods

### 2.1. Participants

The sample consisted of 1521 participants (48% boys and 52% girls) from 9 to 14 years (M = 12.1; SD = 1.3). The sample selection followed an approximately proportional stratified procedure that included 18 primary and lower and upper secondary schools in both urban and rural populations located throughout the Region of Extremadura (Spain). In the urban cases, the schools corresponded to both the center and the periphery of the town, so that the final overall sample would cover diverse socio-economic contexts. Finally, in each of these schools, the year from which to choose the participants was selected at random. The inclusion of urban and rural areas had the objective of covering populations with very different family incomes. In the rural areas selected, the family income level was below the regional average, and approximately half of the participants’ parents had no university studies. In the urban areas, we selected schools located in residential areas, where there is a medium-to-high level of purchasing power, and schools located in humbler neighborhoods where people usually work in low-skilled jobs and where the family income level is medium-to-low.

According to the Regional Education Administration’s data, there were 48,445 pupils of ages between 9 and 14 years enrolled in the Region’s schools. Given this number and taking a confidence level of 95% and an error of 2.5%, in order to be representative, the sample should comprise 1489 subjects. In our case, the sample exceeded that figure with 1521 participants, so that it can indeed be said to be representative.

### 2.2. Instruments

The instruments used for data acquisition were two questionnaires. The first was an adaptation of others questionnaires which were aimed at studying the prevalence of bullying and cyberbullying victims [48,49]. The adaptation was based on integrating into a single instrument the questions aimed at identifying the victims of bullying and of cyberbullying. In total, this first questionnaire consists of 48 items, 26 oriented to off-line contexts, and 22 to on-line contexts. The confirmatory factor analysis (CFA) performed for each of these scales (bullying y cyberbullying) showed a good fit. Bullying scale: χ² = 157.89, *p* < 0.001, CFI = 0.96, TLI = 0.94, RMSEA = 0.039. Cyberbullying scale: χ² = 196.01, *p* < 0.001, CFI = 0.97, TLI = 0.96, RMSEA = 0.023. The forms of aggression contemplated for the analysis of off-line contexts were grouped into six categories: exclusion, verbal abuse, direct physical aggression, indirect physical aggression, threats, and sexual harassment. The coefficient of reliability (Cronbach’s alpha) reached in each of the six categories was in the range of 0.71 to 0.84. In the case of the on-line contexts, eight categories were considered: Insults (including homophobia), threats (including blackmail), spreading false rumors (in order to harm the victim’s reputation, damage friendships, or humiliate), exclusion (from contact lists, social networks, etc.), impersonation (another’s identity is used to send or post material of insulting, inappropriate, or embarrassing content in order to damage the victim’s reputation or friendships), sexting, publication of denigrating images or videoclips, and recording and disseminating physical aggressions. The coefficient of reliability (Cronbach’s alpha) reached in each of the eight categories was in the range of 0.73 to 0.88. For the study of each of these categories, questions were included relating to the use of different technological resources (i.e., chats, e-mails, forums, smartphones, and social networking) through which these abuses were carried out. The participants had to answer these questions (both the bullying and the cyberbullying items) indicating the frequency with which they had suffered each category of abuse during the preceding three months. The scale used consisted of four values: Never, once or twice, once a week, and several times a week.

In accordance with the most consensual position that we found regarding the number of forms of abuse a victim had to suffer to be considered a polyvictim, in the present study, we shall identify as polyvictims the participants who felt they had been subjected to four or more types of abuse, regardless of the context in which these had occurred. Regarding the intensity or frequency of the aggressions suffered, and in accordance with the “repetition of aggression” criterion present in the conceptualization of both bullying and cyberbullying, we shall only consider as victims those participants who had been subjected to continued abuse. In our case, the instrument used quantifies the victims and polyvictims who have suffered abuse in the last three months with different degrees of frequency. Given that this is a very short time interval, we consider both those who have suffered abuses “often” (one or twice) and those who have suffered them “very often” (once a week and several times a week) to come within the category of polyvictim.

The second instrument was the questionnaire published by Bandura [30]. This reveals the moral disengagement mechanisms that the participants apply to themselves. They are grouped into eight categories: Moral justification, euphemistic language, advantageous comparison, displacement of responsibility, diffusion of responsibility, distortion of consequences, attribution of blame, and dehumanization. The presence of one or a combination of these categories provides information about the processes of moral control and the selective location of moral disengagement as a function of the focus of the behavior, the agent of the action, the outcomes that flow from the behavior, and the recipient of the actions. However, since these polyvictims may be subject to multiple abuses in both face-to-face and cybernetic contexts, we adapted this scale by first considering for each item situations corresponding to the face-to-face context, and then adding to the same item another similar situation corresponding to the cybernetic context. To perform these adaptations, we took as referents the adjustments that others researchers made to the Bandura questionnaire [36,50,51]. Examples of each of the eight categories would be the following: Moral justification (e.g., “It is all right to cyberbullying others when they have treated you unfairly”), Euphemistic language (e.g., “Re-tweeting false messages about someone is just a form of fun or joking”), Advantageous comparison (e.g., “It’s okay to email a mean message to another kid because posting it on Facebook for everyone to see is worse”), Displacement of responsibility (e.g., “Kids who cyberbully others because their friends make them do it should not be blamed for what they do”), Distortion of consequences (e.g., “Cyberbullying doesn’t really hurt anyone”), Attribution of blame (e.g., “If people give out their passwords to others, they deserve to be cyberbullied”), Dehumanization (e.g., “It’s okay to spread humiliating messages and videos of someone who behaves like an imbecile”). A five-value ordinal scale was used to indicate the degree of agreement with each item, ranging from “strongly disagree” to “strongly agree”. Before its definitive application, the questionnaire was subjected to a confirmatory factor analysis to verify the existence of the eight factors (disengagement mechanisms) and their associated items. The results showed the fit to be adequate: χ² = 248.65, *p* < 0.001, CFI = 0.95, TLI = 0.96, RMSEA = 0.043. The coefficient of reliability (Cronbach’s alpha) reached in each of the eight categories was in the range 0.67 to 0.83.

### 2.3. Procedure

Prior to the distribution of the questionnaires to the participants, both the research objectives and the procedure, instruments and techniques used were checked and approved by the Bioethics and Biosafety Committee of University of Extremadura (Spain). Additionally, the parents’ approval was required (as the study was dealing with minors) as was that of the Regional Education Administration (from both the school inspectors and the schools’ head teachers). In the case of the parents, they were sent a letter describing the nature of the investigation and the mechanisms used to guarantee the anonymity and confidentiality of their children’s responses. This letter was accompanied by a written informed parental consent that they were to send back to the school if they wanted their child to be part of the study sample. In the case of the Education Administration, obtaining approval consisted of two phases. In the first, a detailed report of the objectives and methods of the investigation was sent to the Inspection Service of the Regional Government, together with the ethical principles conforming it. Approval of this report allowed access to the Region’s schools for distribution of the questionnaires. The second phase required acceptance on the part of the selected schools’ management teams to facilitate the choice of classrooms and access to them during school hours.

Once all the favorable permissions had been obtained, the questionnaires were handed out by the researchers who remained in the classrooms while the children and adolescents completed them, and then gathered the completed questionnaires in. In this way, the confidentiality of the data was guaranteed, and any doubts the respondents had about any term or wording in the items could be answered.

## 3. Data Analysis

Based on the identification and study of the prevalence of victims and polyvictims, once the criteria of normality, homoscedasticity, and randomness had been ensured, we applied an analysis of variance (ANOVA) to the data, this being a generalization of Student’s t-test for two independent samples, in order to determine whether having been subjected to more than four different types of abuse implies the use of moral disengagement mechanisms different from those resorted to by the victims. A hierarchical regression analysis was used to determine the predictive effect of the levels of moral disengagement on the polyvictimization processes. Statistical calculations were done using the software package SPSS 22.0 (IBM, Armonk, NY, USA).

## 4. Results

### 4.1. Identification of Polyvictims

The prevalence results showed the number of victims (*n* = 257) and polyvictims (*n* = 249) to overall be very similar. We excluded from these two subsamples (victims and polyvictims) those cases that can be identified with the role of bully/victim, given that the use of moral disengagement mechanisms could vary with respect to that of victims or polyvictims. With respect to the types of context the abuses come from, the results show that the prevalence in cybernetic scenarios far exceeds that in face-to-face scenarios. Additionally, there was confirmation of a continuity between the off-line and on-line contexts in victimization processes. The prevalence data showed a striking coexistence of the bullying and cyberbullying phenomena. In particular, there were more victims and polyvictims in this convergence of contexts than in each separately (Table 1).

### 4.2. Moral Disengagement as a Predictive Variable of Polyvictimization Processes

A descriptive analysis showed the variability of moral disengagement mechanisms used by the different groups of polyvictims, taking into account the origin of the aggressions they had received (Table 2). Independently of the context of the abuse suffered, the moral disengagement mechanisms most used by polyvictims are moral justification, use of euphemistic language, advantageous comparison, and distortion of consequences.

The lowest levels of moral disengagement corresponded to the use of mechanisms related to dehumanization of the victim and blaming others (Table 2). The highest levels showed that the polyvictims’ moral disengagement is basically founded on two levels: Control of the behavior, and control of the result.

The results of the analysis of variance (ANOVA) showed differences in the use that victims and polyvictims make of the moral disengagement mechanisms that are fundamentally related to behavior locus and outcome locus (Table 3). In general terms, polyvictims score higher than victims in practically all the mechanisms. Hence, the difference between the two groups in the overall moral disengagement score is also significant, being higher in polyvictims (*p* < 0.05).

Knowledge of the presence of levels of moral disengagement in polyvictims justifies the analysis of its predictive value for the conversion from the role of victim or witness to that of a polyvictim.

The hierarchical regression analysis consisted of six models that covered the different levels of moral disengagement, as well as the independent variables considered in this study. Model 1 includes the variables gender (0 male, 1 female) and context (0 bullying, 1 cyberbullying, 2 bullying/cyberbullying). Model 2 adds the variable ‘behaviour locus’. Model 3 adds the variable ‘outcome locus’. Model 4 adds the variable ‘agency locus’ to the previous set. Model 5 adds the variable ‘locus of the recipients’. And finally, model 6 adds ‘moral disengagement’ to all the foregoing variables. The results indicate that the general model that predicts polyvictimization is notably significant (F = 18.64, *p* < 0.01). In the final model, the effects of context, behavior and outcome locus, and moral disengagement emerged as being significant predictors. It was interesting that moral disengagement was also significant despite agency locus and locus of the recipients not being so (Table 4). The gender variable did not prove to have predictive value in any of the models. The variables ‘behaviour locus’ and ‘outcome locus’ were more significant when ‘agency locus’ and ‘locus of the recipients’ were included in the models.

## 5. Discussion

Studies of moral disengagement have traditionally been linked to understanding the possible causes that motivate aggressors to abuse their peers [30,31,50,52]. In recent years, the study of moral variables has also extended to the figure of witness and defender of the victim with the aim of suitably adjusting violence prevention programs for children and teenagers [33,46,53,54]. However, there have been very few studies addressing the link between moral disengagement and victimization. Sometimes this link is even treated as a secondary topic, thus hindering the establishment of comparative studies or the consideration of complementary variables [42,43,55]. We think that one of the most relevant contributions of the present work is that of its thorough analysis of the processes of moral disengagement in victims, and the influence these have on the persistence of their role as victims and their conversion into polyvictims.

With regard to the detection of polyvictims, this study has covered three different scenarios that yield different prevalence data. The results showed that bullying in off-line contexts is less frequent than in on-line contexts. The possibilities the aggressors have of hiding their identity with the use of technological tools, and of increasing the harm done to the victim who feels defenseless by not knowing who their aggressor is or how to stop the rapid and massive dissemination of the abuses they are suffering, could explain why the forms of aggression are undergoing a transfer from off-line to on-line contexts [56]. In our results, fewer than 1% of the participants were definable as polyvictims of bullying, but the proportion rose to more than 3% when dealing with polyvictims of cyberbullying.

However, the coexistence of two different worlds in which we operate in parallel, the off-line and the on-line worlds, has led to a new profile of victim resulting from the continuity of aggressions experienced in physical contexts passing into cybernetic contexts [57]. The child or adolescent who is subjected to abuse by their peers in school probably also suffers abuse through technological media, thus becoming a cybervictim [58]. Experiencing abuse in the two contexts means that they face more forms of aggression that they might otherwise have been subjected to, and hence resulting in an increase in the prevalence of bullying and cyberbullying polyvictims.

Child and teenage victims of peer abuse experience different levels of moral disengagement which make it difficult for them to deal with their victimization situations, and which could turn them into potential polyvictims not only in either an off- or an on-line scenario, but in both simultaneously. Among other factors, the ease with which aggressions spread in the cybernetic world and the possibilities of anonymity the aggressors make use of when abusing other people without suffering reprisals lead to an increase in the number of abuses suffered, and consolidate cyberspace as being the main instrument used to carry out abuse [58]. These distortions in the development of morality may equally be leading to a greater number of forms of aggression suffered, so that the victim becomes a polyvictim.

The positive relationship found between moral disengagement and the condition of victim or polyvictim, in both off-line and on-line contexts and contexts in which the two coexist, reflects a situation of victims resorting to self-incrimination as a cognitive resource allowing them to justify the aggressions they have been subjected to.

The results of this study show that, as the levels of disengagement increase, the prevalence of polyvictims significantly increases relative to the number of victims. Likewise, there is a greater variety of moral disengagement mechanisms to which polyvictims resort, evidence for the enormous tension they suffer, and their need to activate a multitude of mechanisms to release that tension and put a brake on the self-attacks they make on their own self-esteem in being unable to confront the abuse they suffer, or the cowardice of not doing so for fear of feeling excluded or of suffering new attacks as revenge [40].

The analysis of the moral disengagement mechanisms that the different categories of poyvictims use revealed that the most commonly used were moral justification, distortion of consequences, use of euphemistic labeling, and minimization of the consequences. All of these are oriented towards lessening the importance of the actions that they have had committed against them and of the consequences deriving from those actions. Polyvictims try to camouflage or distort the possible intentionality of the aggressor who is attacking them as a form of emotional self-protection [59]. While these distortions may at first play the role of defence mechanisms, they nevertheless contribute to perpetuating the role of victim and favor the processes of polyvictimization.

Finally, the results indicate that the extent to which children and teenagers who are subjected to processes of victimization justify the aggressor’s behavior (behavior locus) or minimize the damage the abuse they received is causing them (locus of the recipients) means that there is a very strong chance of their becoming polyvictims.

Studies of moral disengagement have mostly been centered on the role of the aggressor. The present study opens the way to new lines of research focused on the victim, and on the influence that moral variables have on overcoming, or, on the contrary, multiplying and perpetuating their victimization experiences. All this will enable the activation of new preventive and intervention measures that can be more effective, and provide cognitive, emotional and social tools that allow the victims to face their situations of helplessness, fear, and attacks on their self-esteem, among other factors.

## 6. Conclusions

The great variety of public health policies, programs, and measures designed to prevent bullying and cyberbullying are not enough, or not effective enough, to eradicate these problems in a population as defenceless as children or teenagers. The access to ever more screens, the enormous number of hours that children and teenagers spend connected to the Internet without parental control, the diversification of their social networks (more in cybernetic than in face-to-face contexts) are promoting new forms of relationship and interaction that also bring with them new forms of conflict and aggression. The immaturity and lack of cognitive, emotional, and instrumental resources of these populations make it difficult for them to successfully confront the abusive and violent situations that they may experience. Instead, they tend to activate moral disengagement mechanisms that free them from the social and personal pressure they suffer when they feel attacked. The fear of exclusion, possible reprisals, or ridicule, as well as the avoidance of feelings related to helplessness, cowardice, and inability, inter alia, can lead them to conceal the aggressions they are suffering by self-justifying them as facts that lack seriousness and intentionality and that do not cause them any irreparable damage. This situation in turn means that there will be no action from support networks since they will be unaware of the aggressions the victims are suffering and the damage being caused. This places the victims in a situation of even greater defencelessness that makes their conversion from victims to polyvictims more likely. Undoubtedly, exposure to abuse that is continuous, of different types, and coming from different contexts has enormous repercussions for the mental health of those involved. However, it must also be perceived as a public health problem given the lack of effective tools to combat the situations of helplessness that the polyvictims experience.

## 7. Limitations

This work has some limitations. First, it was a cross-sectional study, so that there has to be caution in making any generalization of the results or in determining any causal and predictive relationships. Secondly, the analyses did not take age into account as a variable. While the ages of the participants cover an interval that is not very broad (9–14 years), the evolutionary moment at which these participants find themselves may have had some sort of influence on the results.

## Figures and Tables

**Table 1:** Prevalence of victims and polyvictims depending on the contexts to which the aggressions belong.

Number of Abuse Modalities Suffered	Victims of Bullying	Polyvictims of Bullying	Victims of Cyberbullying	Polyvictims of Cyberbullying	Victims of Bullying and Cyberbullying	Polyvictims of Bullying and Cyberbullying
Less than 4	67	-	75	-	115	-
4	-	6	-	39	-	97
5	-	1	-	11	-	56
6	-	-	-	3	-	28
7	-	-	-	-	-	5
8	-	-	-	-	-	2
9	-	-	-	-		1
Total	67	7	75	53	115	189

**Table 2:** Mean scores of moral disengagement mechanisms.

Mechanisms of Moral Disengagement	Polyvictims of Bullying	Polyvictims of Cyberbullying	Polyvictims of Bullying and Cyberbullying
	Mean (sd)	Mean (sd)	Mean (sd)
Moral justification	3.14 (0.52)	3.41 (0.67)	3.28 (0.61)
Euphemistic language	2.90 (0.66)	3.31 (0.73)	3.16 (0.70)
Advantageous comparison	2.82 (0.70)	3.19 (0.77)	2.99 (0.71)
Distortion of consequences	2.96 (0.70)	3.58 (0.72)	3.22 (0.69)
Diffusion of responsibility	2.02 (0.67)	2.18 (0.64)	1.86 (0.58)
Displacement of responsibility	1.89 (0.70)	1.94 (0.66)	2.11 (0.62)
Dehumanization	0.97 (0.63)	1.07 (0.49)	1.10 (0.50)
Attribution of blame	1.27 (0.51)	1.34 (0.58)	1.31 (0.54)

**Table 3:** Comparison in the use of moral disengagement mechanisms between victims and polyvictims.

Moral Disengagement Mechanisms	Victims	Polyvictims	F	Cohen’s D
Moral justification	2.01 (0.17)	3.14 (0.19)	11.77 **	0.95
Euphemistic language	2.74 (0.14)	2.93 (0.15)	3.20	0.55
Advantageous comparison	1.90 (0.12)	2.97 (0.15)	9.14 *	0.97
Behavior locus	2.11 (0.18)	2.98 (0.16)	8.29 *	0.93
Distortion of consequences	2.32 (0.17)	3.24 (0.17)	10.28 **	0.94
Outcome locus	2.32 (0.17)	3.24 (0.17)	10.28 **	0.94
Diffusion of responsibility	1.96 (0.14)	2.15 (0.15)	4.68	1.31
Displacement of responsibility	1.64 (0.15)	2.23 (0.14)	7.05 *	0.90
Agency Locus	1.80 (0.15)	2.11 (0.15)	3.78	0.72
Dehumanization	0.94 (0.12)	1.01 (0.14)	0.98	0.26
Attribution of blame	1.57 (0.13)	1.29 (0.15)	2.03	0.71
Locus of the recipients	1.28 (0.15)	1.14 (0.16)	1.34	0.41
Moral disengagement	2.13 (0.17)	2.91 (0.19)	6.89 *^,^^1^	0.91

^1^ Standard error are in parentheses. Degrees of freedom for each analysis = 2. * *p* < 0.05, ** *p* < 0.01.

**Table 4:** Hierarchical regressions.

Variables	Polyvictimization					
	Model 1 β ^1^ (t)	Model 2 β ^1^ (t)	Model 3 β ^1^ (t)	Model 4 β ^1^ (t)	Model 5 β ^1^ (t)	Model 6 β ^1^ (t)
Gender	0.16 (3.21)	0.16 (3.00)	0.14 (2.89)	0.13 (2.52)	0.09 (2.15)	0.08 (1.89)
Context	0.28 (7.54) *	0.31 (8.02) *	0.29 (7.88) *	0.27 (7.36) *	0.27 (7.36) *	0.29 (7.81) *
Behavior locus	-	0.47 (11.54) **	0.43 (10.73) **	0.36 (8.55) *	0.31 (7.98) *	0.39 (9.14) **
Outcome locus	-	-	0.34 (8.97) **	0.28 (7.76) *	0.26 (7.35) *	0.32 (8.75) **
Agency locus	-	-	-	0.19 (4.13)	0.14 (2.87)	0.12 (2.56)
Locus of the recipients	-	-	-	-	0.12 (2.26)	0.15 (2.87)
Moral disengagement	-	-	-	-	-	0.36 (8.84) **
R^2^	0.09	0.17	0.26	0.31	0.28	0.35
ΔR^2^		0.09	0.12	0.15	0.10	0.17 ^1^

^1^ Represents standardized coefficients. * *p* < 0.05, ** *p* < 0.01.
